# Distinct predictive impact of FISH abnormality in proteasome inhibitors and immunomodulatory agents response: redefining high‐risk multiple myeloma in Asian patients

**DOI:** 10.1002/cam4.1340

**Published:** 2018-01-29

**Authors:** Ja Min Byun, Dong‐Yeop Shin, Junshik Hong, Inho Kim, Hyun Kyung Kim, Dong Soon Lee, Youngil Koh, Sung‐Soo Yoon

**Affiliations:** ^1^ Department of Internal Medicine Seoul National University College of Medicine Seoul National University Hospital Seoul Korea; ^2^ Department of Internal Medicine Seoul Metropolitan Government Seoul National University Boramae Medical Center Seoul Korea; ^3^ Biomedical Research Institute Seoul National University Hospital Seoul Korea; ^4^ Cancer Research Institute Seoul National University Hospital Seoul Korea; ^5^ Department of Laboratory Medicine Seoul National University College of Medicine Seoul Korea

**Keywords:** Asian, immunomodulatory drugs, in situ hybridization, multiple myeloma, proteasome inhibitors

## Abstract

For risk‐adaptive therapeutic approaches in multiple myeloma (MM) treatment, we analyzed treatment outcome according to in situ hybridization (FISH) profiles to investigate the prognostic and predictive values of structural variations in a large series of Asian population. A total of 565 newly diagnosed patients with multiple myeloma between January 2005 and June 2015 were evaluated. FISH results showed del(17p13) in 8.8% (29/331), del(13q14) in 35.5% (184/519), *t*(14;16) in 2.5% (8/326), *t*(4;14) in 27.9% (109/390), IgH rearrangement in 47.7% (248/520), and +1q21 in 40.8% (211/517). The presence of del(17p13), IgH rearrangement, and *t*(14;16) was associated with worse overall survival. Interestingly, however, the presence of *t*(4;14) conferred little prognostic impact. Treatment‐specific analysis revealed the presence of del(17p13), *t*(14;16), IgH rearrangement, and trisomy 1q21 was predictive of unsatisfactory response to bortezomib. On the other hand, patients with del(13q14) and del(9p21) were less likely to benefit from lenalidomide. Autologous stem cell transplantation (autoSCT) was less effective in patients with del(17p13), *t*(14;16), and trisomy 1q21. Predictive values of del(17p13) and *t*(14;16) to bortezomib and autoSCT are seemingly universal, but predictive marker del(13q14) and del(9p21) for lenalidomide response appears ethnicity‐specific. Thus, FISH profiles in MM treatment should be interpreted with regards to patient's ethnicity.

## Introduction

Multiple myeloma (MM) represents a considerable clinical challenge as both the number of patients 1 and the treatment cost have been rising 2, 3, 4. Once believed to be a homogeneous disease entity, MM now epitomizes heterogeneous genomic evolution and mutational profiles with varying clinical course and response to treatment 5, 6. The initiating genetic event is believed to involve recurrent translocations at the immunoglobulin heavy‐chain (IgH) locus on chromosome 14q32, deletions of chromosome 13, and dysregulated expression of cyclin D genes. Additionally, deletion of 17p13, c‐myc translocations, and gain of chromosome 1q21 are associated with disease progression and transformation. As such, fluorescence in situ (FISH) is integral part of MM diagnosis to detect the widespread structural variations 7, 8.

In attempts to identify predictive and prognostic markers for MM, various groups have proposed different risk stratification methodologies based on molecular, cytogenetic, and clinical data (https://www.nccn.org/professionals/physician_gls/pdf/myeloma.pdf) 6, 9, 10, 11, 12, 13. For example, the International Myeloma Working Group and the Mayo group established a consensus that FISH testing for *t*(4;14), *t*(14;16), and del(17p13) should be carried out for all patients to identity high‐risk disease 6, 9, 13. Meanwhile, the Medical Research Council Myeloma IX trial suggested performing additional FISH analysis for trisomy 1q and *t*(14;20) to further aid in risk stratification 10 and current NCCN guideline recommends FISH testing for deletion 13, del(17p13), *t*(4;14), *t*(11;14), *t*(14;16), and trisomy 1q21 (https://www.nccn.org/professionals/physician_gls/pdf/myeloma.pdf). As such, the prognostic predictive value of each chromosomal aberration by FISH varies per reporting group; thus, large‐scale data analysis is imperative to accurately explore the impact of specific FISH abnormality.

Another point to note is the disparity in disease incidence and clinical behavior among patients from different ethnic and geographical backgrounds 14, 15 and the apparent lack of comprehensive Asian data. Available data from China 16, 17 and Korea 18, 19 reported the prognostic impact of del(17p13) and chromosome 13 deletion on overall survival (OS), and the significance of del(17p13), *t*(4;14), and trisomy 1q21 on progression‐free survival (PFS), delineating the racial diversity between Caucasians and East Asians. In this clinical picture, adopting risk stratification model based heavily on Caucasian population might be suboptimal for individualized treatment.

Considering the rapidly increasing MM incidence in Asia, we sought to establish risk stratification schema incorporating both conventional cytogenetics and FISH data for continuous optimization of MM treatment based on one of the biggest cohort‐based studies. We also investigated FISH abnormalities in relation to treatment response to newer therapeutic agents, in hopes of establishing predictive markers for different treatment. Korean population was selected for this study, because Korea has a sole public medical insurance system that is mandatory and covers approximately 98% of the overall Korean population 20 and the range of coverage is strictly controlled; thus, the general MM treatment algorithm is relatively uniform throughout the population.

## Methods

### Study design and subjects

**Figure 1:**
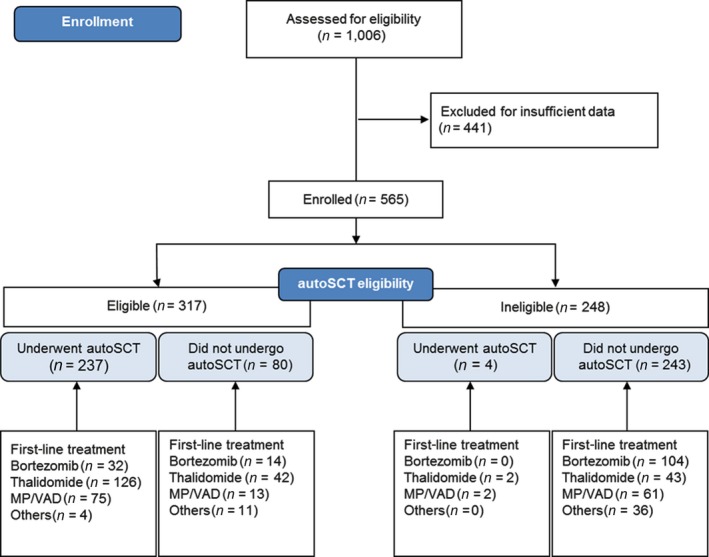
Patient triage flow. AutoSCT, autologous stem cell transplantation; MP, melphalan–prednisone; VAD, vincristine–doxorubicin–dexamethasone.

This study was carried out at Seoul National University Hospital, a tertiary academic center and one of the hospitals with highest volume of patients in Korea. During the period between January 2005 and June 2015, 1006 newly diagnosed MM patients were retrospectively identified. Adult patients, defined as 18 years old or older, were included, while cases with smoldering myeloma, monoclonal gammopathy of unknown significance, and solitary plasmacytoma were excluded in the first place. After excluding additional 441 patients for insufficient data, a total of 565 patients with a complete set of molecular information were evaluated for their demographic, laboratory, and clinical data (Fig. 1). Autologous stem cell transplantation (autoSCT)‐eligible patients were defined as those under the age of 65 years according to national insurance coverage restrictions.

This study was conducted according to the Declaration of Helsinki and was approved by the institutional review board of Seoul National University Hospital (IRB No. H‐1602‐033‐739).

### Conventional cytogenetic analysis and FISH

Cytogenetic studies were performed at our center, whose satisfactory performance was monitored and censored by a national quality assurance system. Unstimulated bone marrow cells obtained at MM diagnosis were cultured for 24 h; then, karyotype was analyzed using the standard G‐banding technique. The karyotypes were constructed and chromosomal abnormalities were reported according to the International System for Human Cytogenetic Nomenclature (ISCN 2009) 21.

Interphase FISH study was performed on myeloma cells from the bone marrow samples at diagnosis according to the manufacturers’ instructions. As there are no standard MM FISH probes used in Korea, seven commercially available FISH probe sets were used. These included *IgH* dual‐color, break‐apart rearrangement probe; *TP53* SpectrumOrange probe; D13S25 (13q14.3) SpectrumOrange probe; *IgH/MAF* dual‐color, dual‐fusion translocation probe; *IgH/FGFR3* dual‐color, dual‐fusion translocation probe; 1q21/8p21 dual‐color probe; and CDKN2A (9p21, p16) SpectrumOrange/CEP9 SpectrumGreen probe (Abbott Diagnostics, Abbott Park, IL).

### Statistical analysis

Differences between groups were assessed using a Student's *t*‐test or one‐way analysis of variance for continuous variables and Pearson chi‐square test for categorical variables, as indicated. The overall survival (OS) and progression‐free survival (PFS) curves were estimated using the Kaplan–Meier method. OS was defined as the time from MM diagnosis to death from any cause. PFS of bortezomib or lenalidomide was defined as the duration from the start of bortezomib or lenalidomide to disease progression or death. PFS of autoSCT was defined as the duration from the date of transplantation to disease progression requiring treatment or death. If patients survived without death or progression, survival was censored at the latest date of follow‐up when no death or progression was confirmed, and data available up to June 2016 were used. Univariate and multivariate proportional hazards regression models were used to identify independent risk factors of PFS and OS by means of log‐rank tests and Cox proportional hazards models, respectively. A stepwise backward procedure was used to construct a set of independent predictors of each end point. All predictors achieving a *P* value below 0.10 were considered and sequentially removed if the *P* value in the multiple models was above 0.05. All data were analyzed using the Statistical Package for the Social Sciences software (IBM^®^ SPSS^®^ Statistics, version 22.0 Chicago IL). *P* values of <0.05 were considered statistically significant.

## Results

### Patient characteristics

The baseline patient characteristics of the 565 patients are shown in Table 1. The median age was 63 years (18–92 years), and there were 309 males (54.7%). The proportion of patients at International Staging System (ISS) stage III was 32.4%, while those at Revised International Staging System (R‐ISS) stage III was 50.1%. Among the enrolled patients, 42.7% underwent autoSCT. For those undergoing autoSCT, the most common induction therapy used was thalidomide based (128/241, 53.1%), followed by cytotoxic chemotherapy based (77/241, 32.9%). Bortezomib‐based induction was used in 46 patients (19.1%), and none received lenalidomide as induction for autoSCT (Table 1 and Fig. 1). All of the patients receiving conventional chemotherapy as first‐line treatment were subsequently exposed to either proteasome inhibitors and/or IMiDs in subsequent treatments.

**Table 1:** Baseline characteristics of 565 enrolled patients

Characteristics	*N* (%)
Age	
Median (years, range)	63 (18–92)
<65 years	317 (56.1)
≥65 years	248 (43.9)
Sex	
Male	309 (54.7)
Performance status	
ECOG 0–1	307 (54.4)
ECOG ≥2	252 (44.6)
Missing	6 (1.0)
Ig type	
IgG / A / Others	276 (48.8) / 102 (18.1) / 23 (4.0)
Light chain	164 (29.0)
Light chain	
Kappa / Lambda	304 (53.8) / 261 (46.2)
Missing	0
ISS	
I/II/III	155 (27.5) /190 (33.6) /183 (32.4)
Missing	37 (6.5)
R‐ISS	
I/II/III	39 (6.9) / 251 (38.1) / 283 (50.1)
Missing	28 (5.0)
Azotemia at MM diagnosis	
Creatinine >2 mg/dL	104 (18.4)
Creatinine ≤2 mg/dL	461 (81.6)
Treatment	
autoSCT	241 (42.7)
Thalidomide exposure	322 (57.0)
Bortezomib exposure	398 (70.4)
First line	150
Second line and beyond	248
Lenalidomide exposure	145 (25.7)
First line	8
Second line	24
Third line and beyond	113

ECOG, Eastern Cooperative Oncology Group; DSS, Durie–Salmon staging; ISS, International Staging System; R‐ISS, Revised International Staging System; MM, multiple myeloma; SD, standard deviation; autoSCT, autologous stem cell transplantation.

### Conventional cytogenetics and FISH abnormalities

The frequency of each FISH panel used varied (Table 2). IgH rearrangement was tested most often (520/565, 92.0%) and *t*(14;16) least often (326/565, 57.7%). Overall, there were 277 (49.0%) patients with all seven FISH panels. FISH results showed del(17p13) in 8.8% (29/331), del(13q14) in 35.5% (184/519), *t*(14;16) in 2.5% (8/326), *t*(4;14) in 27.9% (109/390), IgH rearrangement in 47.7% (248/520), trisomy 1q21 in 40.8% (211/517), and del(9p21) in 2.2% (11/505) of cases.

**Table 2:** FISH abnormalities

	Tested	Positive (%)	Alone (%)	Combination (%)		
				Two	Three	Four or more
del(17p13)	331	29 (8.8)	7 (24.1)	3 (10.3)	4 (13.8)	15 (51.7)
del(13q14)	519	184 (35.5)	19 (10.3)	59 (32.1)	72 (39.1)	34 (18.5)
*t*(14;16)	326	8 (2.5)	1 (12.5)	1 (12.5)	2 (25.0)	4 (50.0)
*t*(4;14)	390	109 (27.9)	63 (25.4)	2 (1.8)	19 (17.4)	25 (23.0)
IgH rearrange	520	248 (47.7)	68 (27.4)	66 (26.6)	79 (31.9)	35 (14.1)
+1q21	517	211 (40.8)	42 (19.9)	66 (31.3)	70 (33.2)	33 (15.6)
del(9p21)	505	11 (2.2)	5 (45.5)	1 (9.1)	3 (27.3)	2 (18.2)

FISH, fluorescence in situ hybridization; IgH rearrange, IgH rearrangement.

### FISH abnormalities and treatment response

**Figure 2:**
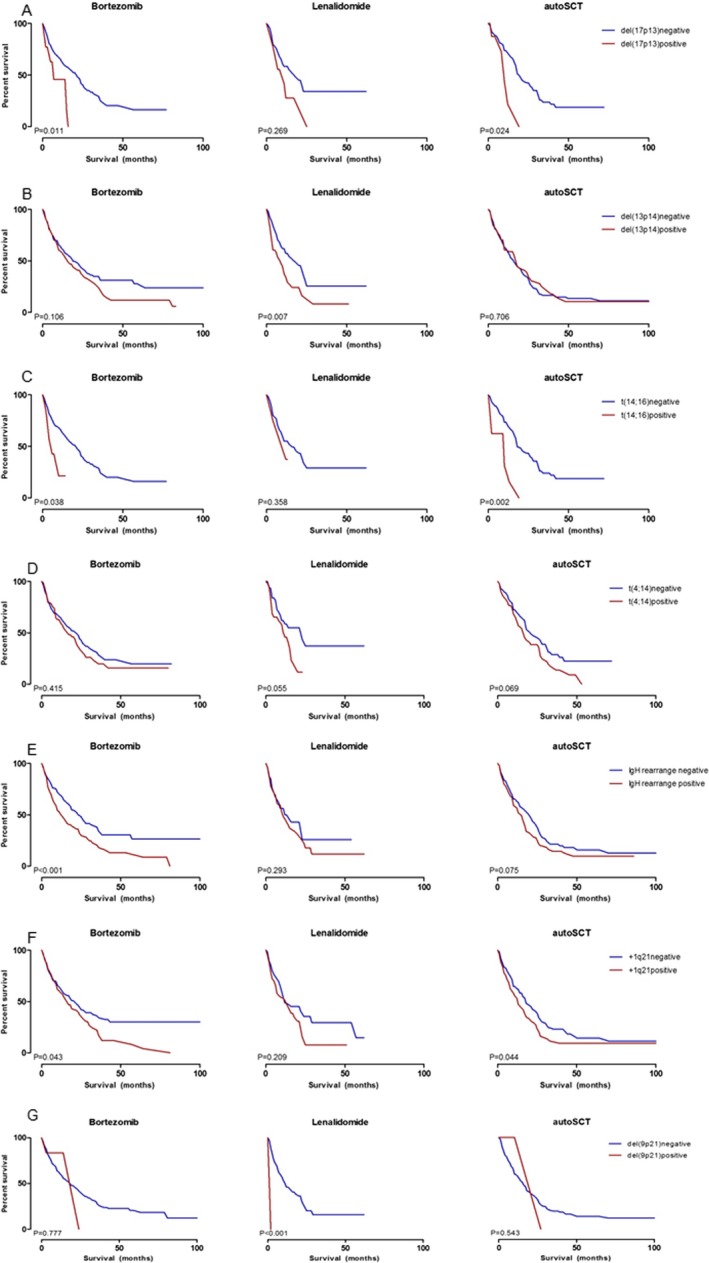
Progression‐free survival (PFS) after bortezomib, lenalidomide treatment, and autologous stem cell transplantation (autoSCT) according to different FISH abnormalities. (A) PFS according to del(17p13) status; (B) PFS according to del(13q14) status; (C) PFS according to *t*(14;16) status; (D) PFS according to *t*(4;14) status; (E) PFS according to IgH rearrangement status; (F) PFS according to trisomy 1q21 status; (G) PFS according to del(9p21) status.

Table 3 and Figure 2 represent treatment response to bortezomib and lenalidomide, regardless of treatment timing, according to different FISH status. The presence of del(17p13) seemed to decrease bortezomib response (Table 3), but the difference did not reach statistical significance. However, the presence of del(17p13) was associated with shorter PFS to bortezomib (median PFS 27 months for del(17p13)‐negative group versus 9 months for del(17p13)‐positive group, *P *=* *0.011) (Fig. 2A). Lenalidomide response was not altered according to del(17p13) status. The presence of del(17p13) was associated with shorter PFS to autoSCT (median PFS 28 months for del(17p13)‐negative group vs. 11 months for del(17p13)‐positive group, *P *=* *0.024).

**Table 3:** Response rates to bortezomib and lenalidomide according to FISH

	Bortezomib					Lenalidomide				
	CR	VGPR	PR	SD/PD	*P* a	CR	VGPR	PR	SD/PD	*P* a
del(17p13)										
Absence	43 (20.2)	47 (22.1)	76 (35.7)	34 (15.9)	0.092	5 (7.0)	5 (7.0)	33 (45.5)	25 (35.3)	1.000
Presence	2 (8.3)	4 (16.4)	9 (37.5)	5 (20.9)		1 (11.1)	0	5 (55.6)	3 (33.3)	
del(13q14)										
Absence	44 (18.9)	50 (21.5)	87 (37.3)	41 (17.6)	0.916	5 (7.2)	8 (11.6)	34 (49.3)	22 (31.9)	0.002
Presence	30 (22.1)	34 (25.0)	41 (30.1)	24 (17.7)		4 (7.7)	2 (3.8)	15 (28.8)	28 (53.9)	
*t*(14;16)										
Absence	45 (20.0)	50 (22.2)	79 (35.1)	35 (15.6)	0.660	6 (7.9)	4 (5.3)	37 (48.7)	26 (34.2)	0.379
Presence	0	0	5 (71.4)	2 (28.6)		0	1 (20.0)	1 (20.0)	3 (60.0)	
*t*(4;14)										
Absence	39 (19.8)	43 (21.8)	73 (37.1)	28 (14.2)	0.401	5 (7.9)	5 (7.9)	30 (47.6)	21 (33.4)	0.505
Presence	14 (16.5)	23 (27.1)	26 (30.6)	15 (17.7)		2 (5.4)	6 (16.2)	13 (35.1)	15 (40.5)	
IgH rearrange										
Absence	44 (23.8)	43 (23.2)	65 (35.1)	25 (13.5)	0.027	6 (10.5)	4 (7.0)	22 (38.6)	24 (42.1)	0.664
Presence	29 (15.6)	43 (23.1)	63 (33.9)	41 (22.0)		3 (4.5)	6 (9.0)	26 (38.8)	30 (44.8)	
+1q21										
Absence	41 (19.2)	47 (22.1)	77 (36.2)	37 (17.4)	0.982	4 (6.6)	8 (13.1)	26 (42.6)	20 (32.8)	0.170
Presence	34 (21.8)	39 (25.0)	48 (30.8)	28 (18.0)		5 (8.1)	4 (6.5)	22 (35.5)	31 (50.0)	
del(9p21)										
Absence	71 (20.2)	80 (22.8)	122 (34.8)	60 (17.1)	1.000	8 (7.1)	10 (8.8)	47 (41.2)	46 (40.4)	0.191
Presence	1 (14.3)	4 (57.1)	1 (14.3)	0		0	0	1 (50.0)	1 (50.0)	

FISH, fluorescence in situ hybridization; CR, complete response; VGPR, very good partial response; PR, partial response; SD, stable disease; PD, progressive disease; IgH rearrange, IgH rearrangement.

a *P*‐value for patients with PR or better versus patients with SD or PD.

The presence of del(13q14) was not associated with bortezomib response, but was associated with response to lenalidomide (Table 3). Those with del(13q14) tended to be more refractory to lenalidomide treatment (PR or better vs. SD or PD, *P *=* *0.002). This phenomenon translated to differences in PFS to lenalidomide (median PFS 24 months for del(13q14)‐negative group vs. 13 months for del(13q14)‐positive group, *P *=* *0.007) (Fig. 2B). The response to autoSCT was not altered by the presence of del(13q14).

Although presence of *t*(14;16) did not have any effects on response rates and PFS to both bortezomib and lenalidomide, its presence was associated with shorter PFS after autoSCT (median PFS 28 months for *t*(14;16)‐negative group vs. 9 months for *t*(14;16)‐positive group, *P *=* *0.002) (Fig. 2C).

The presence of *t*(4;14) did not have any effects on response rates to bortezomib and lenalidomide. Its presence did not deter PFS to bortezomib (*P *=* *0.415), lenalidomide (*P *=* *0.055), or autoSCT (*P *=* *0.069) (Fig. 2D).

The presence of IgH rearrangement was a determinant for bortezomib response (PR or better vs. SD or PD, *P *=* *0.027). The presence of IgH rearrangement was also associated with shorter PFS to bortezomib (median PFS −22 months) compared to those without (median PFS 42 months) (*P *< 0.001), but was not associated with PFS to lenalidomide or autoSCT (Fig. 2E).

The presence of trisomy 1q21 was not associated with response rates to bortezomib or lenalidomide, but its presence deterred the PFS to bortezomib (median PFS 43 months for trisomy 1q21‐negative group vs. 22 months for trisomy 1q21‐positive group, *P *=* *0.043) (Fig. 2F). The presence of trisomy 1q21 was also associated with shorter PFS to autoSCT (*P *=* *0.044).

Although del(9p21) had effects on response rates of both bortezomib and lenalidomide, its presence significantly decreased the PFS to lenalidomide (median PFS 18 months for del(9p21)‐negative group vs. 2 months for del(9p21)‐positive group, *P *< 0.001) (Fig. 2G). Its presence had no role on PFS to autoSCT.

Table S1 shows treatment response to thalidomide according to FISH aberration. There was no particular genetic aberration that was predictive or prognostic of thalidomide response.

### Survival

**Figure 3:**
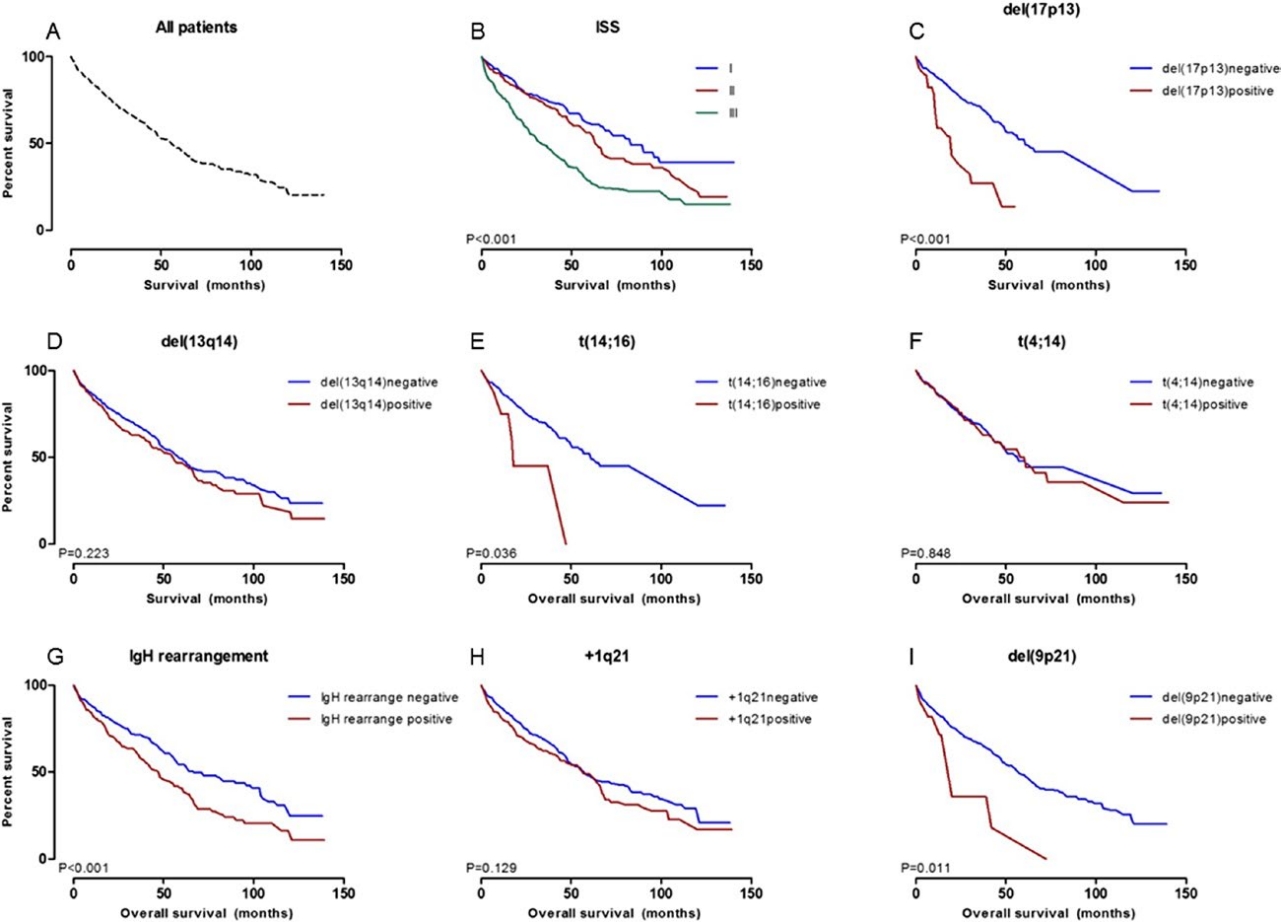
Overall survival (OS). (A) OS of all 565 patients. Median survival 54 months; (B) OS according to International Staging System (ISS); (C) OS according to del(17p13) status; (D) OS according to del(13q14) status; (E) OS according to *t*(14;16) status; (F) OS according to *t*(4;14) status; (G) OS according to IgH rearrangement status; (H) OS according to trisomy 1q21 status; (I) OS according to del(9p21) status.

FISH abnormalities associated with OS were del(17p13), *t*(14;16), IgH rearrangement, and del(9p21) (Fig. 3). On multivariate analyses, ISS stage, lenalidomide exposure, autoSCT, hyperploidy on conventional cytogenetics, and adverse FISH abnormality defined as del(17p13), *t*(14;16), and IgH rearrangement were recognized as important prognostic factors (Table 4).

**Table 4:** Multivariate analyses for overall survival (OS)

Parameters	Univariate		Multivariate	
	HR (95% CI)	*P*	HR (95% CI)	*P*
ISS (III vs. I, II)	2.064 (1.627–2.619)	<0.001	2.434 (1.585–3.739)	<0.001
del(17p13) (Positive vs. Negative)	3.200 (1.919–5.336)	<0.001	3.034 (1.512–6.087)	0.002
*t*(14;16) (Positive vs. Negative)	2.528 (1.027–6.228)	0.044	3.410 (1.067–10.896)	0.038
IgH rearrange (Positive vs. Negative)	1.576 (1.230–2.020)	<0.001	1.857 (1.207–2.857)	0.005
del(9p21) (Positive vs. Negative)	2.408 (1.187–4.886)	0.015	2.414 (0.569–10.247)	0.232
Lenalidomide exposure (Yes vs. No)	0.510 (0.386–0.675)	<0.001	0.413 (0.245–0.696)	0.001
AutoSCT (Yes vs. No)	0.495 (0.390–0.627)	<0.001	0.387 (0.234–0.637)	<0.001

ISS, International Staging System; autoSCT, autologous stem cell transplantation; IgH rearrange, IgH rearrangement; NK, normal karyotype.

On multivariate analysis for bortezomib PFS, IgH rearrangement was identified as significant prognostic factor (HR 1.785, 95% CI: 1.179–2.702, *P *=* *0.006). Line of bortezomib (i.e., first‐line bortezomib, bortezomib after relapse) did not affect the PFS (Table 5). For lenalidomide PFS, del(9p21) (HR 9.360, 95% CI: 2.009–46.302, *P *=* *0.004) and del(13q14) (HR 1.784, 95% CI: 1.083–2.940, *P *=* *0.023) were identified as prognostic factor on multivariate analysis. Use of lenalidomide in first‐line treatment (HR 0.216, 95% HR: 0.064–0.723, *P *=* *0.013) was also associated with better PFS (Table 5).

**Table 5:** Multivariate analyses for progression‐free survival (PFS)

Parameters	Univariate		Multivariate	
	HR (95% CI)	*P*	HR (95% CI)	*P*
For bortezomib PFS				
ISS (III vs. I, II)	1.486 (1.131–1.953)	0.004	1.263 (0.845–1.888)	0.257
del(17p13) (Positive vs. Negative)	2.033 (1.149–3.596)	0.015	1.553 (0.752–3.197)	0.235
IgH rearrange (Positive vs. Negative)	1.662 (1.249–2.211)	<0.001	1.785 (1.179–2.702)	0.006
+1q21 (Positive vs. Negative)	1.332 (1.003–1.770)	0.048	1.052 (0.697–1.588)	0.808
*t*(14;16) (Positive vs. Negative)	2.482 (1.003–6.140)	0.049	1.814 (0.621–5.299)	0.276
Line of bortezomib (1st line vs. others)	0.878 (0.677–1.139)	0.328	NA	NA
For lenalidomide PFS				
ISS (III vs. I, II)	1.274 (0.752–2.157)	0.368	NA	NA
del(9p21) (Positive vs. Negative)	8.720 (2.002–37.986)	0.004	9.360 (2.009–43.602)	0.004
del(13q14) (Positive vs. Negative)	1.882 (1.164–3.045)	0.010	1.784 (1.083–2.940)	0.023
Line of lenalidomide				
First line	0.177 (0.053–0.592)	0.005	0.216 (0.064–0.723)	0.013
Second line	0.457 (0.228–0.919)	0.028	0.447 (0.198–1.013)	0.054
Others	ref	ref	ref	ref

ISS, International Staging System; IgH rearrange, IgH rearrangement; autoSCT, autologous stem cell transplantation; NA, not applicable; ref, reference.

## Discussion

Traditionally believed to be less common among Asians, the incidence of MM in Korea has increased by 10‐fold during the past 20 years 1. More importantly, with the introduction of novel therapeutic agents, MM is incurring more and more medical cost per person. Korea is not an exception, and MM incurred the second highest medical cost per person in 2004 according to public health insurance claim data in Korea. Considering the rapidly aging population and rising treatment cost in association with advances in medical fields, MM poses a cumulative threat to public health, generating substantial economic and social burdens. Although various guidelines recommending optimal approaches to MM treatment are available 9, 10, 11, 13, 21, these guidelines do not take into consideration the racial differences or inconsistent healthcare resource distribution. Also, understanding different efficacy profiles and biology of therapeutic agents is important for optimal combination strategies even in an era where “standard” treatment includes doublet or triplet of novel agents as induction. Thus, using one of the biggest real‐world practice data, we attempted to establish risk stratification specific to Koreans as the first step to implementing personalized risk‐adaptive treatment of relatively under‐represented Asians. Our results showed presence of the del(17p13), IgH rearrangement, and *t*(14;16) on FISH was independent adverse predictors of OS. On the other hand, the presence of *t*(4;14) conferred little prognostic impact. Our data also revealed that patients with del(17p13), *t*(14;16), and IgH rearrangement are less likely to respond to bortezomib, while those with del(13q14) and del(9p21) are less likely to benefit from lenalidomide. AutoSCT was less effective in patients with del(17p13), *t*(14;16), and trisomy 1q21.

One of the major strengths of our study is that we presented response and PFS to novel agents according to different genetic aberrations. In recent years, the treatment landscape of MM has changed drastically with the advent of novel agents such as proteasome inhibitors and immunomodulatory drugs (IMiDS) 22, 23, 24. Hence, we tried to identify predictive FISH marker for bortezomib and lenalidomide response. Among 398 patients exposed to bortezomib at any point during their disease course, we could not identify any FISH marker that could predict better response (Table 3). However, the presence of del(17p13) (*P *=* *0.011), *t*(14;16) (*P *=* *0.038), IgH rearrangement (*P *<* *0.001), and trisomy 1q21 (*P *=* *0.043) was associated with significantly shorter PFS to bortezomib (Fig. 2). On multivariate analysis, IgH rearrangement was significantly associated with bortezomib PFS. Our data agree with previous studies reporting that the use of bortezomib in Caucasian patients with MM cannot overcome the adverse effects of del(17p13) and *t*(14;16) 25, 26, 27.

As for lenalidomide, there were only a handful of patients receiving first‐line lenalidomide, but, as most patients received lenalidomide as salvage treatment option after bortezomib failure, it is justifiable to say that lenalidomide PFS was estimated in a homogeneous population. Our attempts to identify predictive FISH marker for lenalidomide treatment have led to the discovery that the presence of del(13q14) is associated with significantly poorer response to lenalidomide (*P *=* *0.002 for patients with PR or better response vs. patients with SD or PD; Table 3). This also translated into shorter PFS to lenalidomide for patients with del(13q14) compared to those without (*P *=* *0.007, Fig. 2B). Moreover, the presence of trisomy 1q21 and del(9p21) was also associated with shorter PFS to lenalidomide. On multivariate analysis, del(9p21) and del(13q14) were identified as independent predictors for lenalidomide response. This finding disagrees with results of predominantly Caucasian‐based study, which showed lenalidomide could overcome the poor prognosis conferred by del(13q) 28, 29. A follow‐up data from patients receiving first‐line lenalidomide are warranted for more concrete conclusions. Also different from Caucasian data, the presence of del(17p13) was not a determinant for lenalidomide response.

FISH is not always performed for risk stratification of newly diagnosed MM patients, and this led to high rate of exclusion (441/1006, 43.8%) at patient enrollment (Fig. 1). However, it seems that specific FISH markers are definite prognostic markers for overall survival. As previously shown in many studies 6, 11, 22, the presence of del(17p13) remained an adverse prognostic factor in Korean model (HR 4.567, 95% CI: 1.648–12.656, *P *=* *0.003). Although the number of patients was too small to confer definitive significance, we identified the presence of del(9p21) as prognostic factor for OS on univariate analysis. Recently recognized as a new risk locus for MM 30, further investigations are needed to corroborate this finding, as it lost its prognostic power in the multivariate analysis.

All in all, the FISH abnormalities with predictive impact to bortezomib and autoSCT were the prognostic determinants of overall survival. The predictive values of del(17p13) and *t*(14;16) to bortezomib and autoSCT are seemingly universal, but predictive marker for lenalidomide response appears specific to Korean population. The use of lenalidomide is expected to increase considerably in Korea with its approval as first‐line MM treatment as of 27 June 2017. Thus, in near future, the predictive marker of lenalidomide response might also define survival, and accordingly, del(13q14) and del(9p21) deserve clinical attention.

The uniform treatment scheme for MM is both the strength and one of the major pitfalls of our study. Reflecting strict restrictions on use of novel agents, only about 26.5% (150/565) of the patients were able to receive bortezomib as first‐line treatment. There were even fewer patients who were treated with first‐line lenalidomide (8/565, 1.4%), and this was used in clinical trial settings. As first‐line bortezomib is only allowed for autoSCT‐ineligible patients, a survival benefit from first‐line bortezomib was not calculated. However, among 145 patients receiving lenalidomide, lenalidomide was administered after bortezomib failure in all but 8. This, as mentioned before, allowed for nonbiased assessment of lenalidomide efficacy in a rather homogeneous setting. Another limitation to point out is the difference in median follow‐up durations for each FISH probe. The median follow‐up duration for the total cohort was 64 months. The median follow‐up durations for those tested for IgH rearrangement (67 months), trisomy 1q21 (66 months), del(13q14) (68 months), and del(9p21) (66.5 months) were comparable to the median follow‐up duration for the total cohort. Meanwhile, the median follow‐up duration for those tested for *t*(4;14) (39 months), del(17p13) (39.5 months), and *t*(14;16) (39 months) was considerably shorter. Longer follow‐up may be necessary to generate more solid and mature data. Lastly, the lack of information specific to *t*(6;14), *t*(11;14), and *t*(14;20) is also a limitation. Corroboration from larger cohort with more comprehensive data should ensure our study.

## Conclusions

In conclusion, the predictive values of del(17p13) and *t*(14;16) to bortezomib and autoSCT are seemingly universal, but predictive marker del(13q14) and del(9p21) for lenalidomide response appears specific.

## Conflict of Interest

No conflict of interest to disclose.

## Supporting information


**Table S1.** Overall survival (OS) and progression free survival (PFS) of patients receiving thalidomide based treatment as first‐line treatment.
