# Supplementary material for: Distinct predictive impact of FISH abnormality in proteasome inhibitors and immunomodulatory agents response: redefining high‐risk multiple myeloma in Asian patients
Source: Cancer Med. 2018 Jan 29;7(3):831–41. doi: 10.1002/cam4.1340 (PMC5852362; doi:10.1002/cam4.1340)
Supplement: Supplementary file 1 — Table S1. Overall survival (OS) and progression free survival (PFS) of patients receiving thalidomide based treatment as first‐line treatment. [file CAM4-7-831-s001.docx]

**TABLE S1** Overall survival (OS) and progression free survival (PFS) of patients receiving thalidomide based treatment as first line treatment

| **FISH** | **Status** | **OS** | | | | | |  | **PFS** | | | | | |
| --- | --- | --- | --- | --- | --- | --- | --- | --- | --- | --- | --- | --- | --- | --- |
|  |  | Total cohort  N=213 | | Underwent autoSCT  N=128 | | Did not undergo autoSCT  N=85 | |  | Total cohort  N=213 | | Underwent autoSCT  N=128 | | Did not undergo autoSCT  N=85 | |
|  |  | OS^*^ | *P* | OS^*^ | *P* | OS^*^ | *P* |  | PFS-1^**^ | *P* | PFS-1^**^ | *P* | PFS-1^**^ | *P* |
| del(17p13) | Negative | 120 | 0.019 | NR | 0.019 | 27 | 0.459 |  | 22 | 0.123 | 33 | 0.027 | 5 | 0.654 |
|  | Positive | 20 |  | 30 |  | 20 |  |  | 8 |  | 17 |  | 8 |  |
| del(13q14) | Negative | 63 | 0.784 | 90 | 0.721 | 26 | 0.418 |  | 14 | 0.830 | 22 | 0.574 | 7 | 0.059 |
|  | Positive | 66 |  | 72 |  | 17 |  |  | 12 |  | 24 |  | 6 |  |
| t(14,16) | Negative | 120 | 0.232 | NR | 0.012 | 27 | NA |  | 22 | 0.614 | 33 | 0.099 | 5 | NA |
|  | Positive | 47 |  | 47 |  | NA |  |  | 13 |  | 13 |  | NA |  |
| t(4,14) | Negative | 120 | 0.769 | NR | 0.815 | 25 | 0.680 |  | 22 | 0.566 | 33 | 0.172 | 3 | 0.317 |
|  | Positive | 66 |  | 66 |  | 27 |  |  | 21 |  | 24 |  | 11 |  |
| IgH rearrange | Negative | 64 | 0.451 | 72 | 0.892 | 26 | 0.132 |  | 12 | 0.394 | 24 | 0.195 | 7 | 0.235 |
|  | Positive | 65 |  | 115 |  | 19 |  |  | 14 |  | 24 |  | 6 |  |
| +1q21 | Negative | 65 | 0.230 | 99 | 0.669 | 26 | 0.395 |  | 15 | 0.165 | 24 | 0.682 | 7 | 0.109 |
|  | Positive | 63 |  | 69 |  | 16 |  |  | 11 |  | 22 |  | 6 |  |
| del(9p21) | Negative | 63 | 0.069 | 90 | 0.706 | 24 | 0.364 |  | 13 | 0.301 | 24 | 0.946 | 6 | 0.440 |
|  | Positive | 20 |  | 72 |  | 20 |  |  | 4 |  | 32 |  | 4 |  |

Abbreviations: FISH, fluorescence in situ hybridization; autoSCT, autologous stem cell transplantation; NR, not reached; NA, not applicable.

^*^Values represent median overall survival (months)

^**^Values represent median progression free survival (months)
